# Making the Call – Connecting APS Clients to Primary Care

**DOI:** 10.1093/geroni/igaf122.3142

**Published:** 2025-12-31

**Authors:** Ronan Factora, Anna Smith, Camille Smith, Kelly Clemings

**Affiliations:** Cleveland Clinic, Cleveland, Ohio, United States; Case Western Reserve University, Cleveland, Ohio, United States; Case Western Reserve University, Cleveland, Ohio, United States; Division Of Senior and Adult Services, Cleveland, Ohio, United States

## Abstract

Virtual Capacity Evaluations (VCE) are used by the Behavioral Health Unit at Adult Protective Services in Cuyahoga County, Ohio, (APS) to evaluate elder abuse/exploitation/neglect cases where client’s decision making is questioned. The evaluation includes confirming if the client has a primary care physician (PCP) which is important as many have chronic illnesses and observed medication non-adherence. Prior pilot data showed many clients were not connected to a PCP before the VCE (PMID: 36123772), leading to implementation of a recommendation to connect the client with their prior OR new PCP upon VCE completion. This quality improvement project followed up with these clients to see if this recommendation was implemented. Phone contact was made to the responsible party for APS clients who completed VCE from 2023-2024 to confirm if a PCP visit was made 3-6 months following VCE completion. Thirty-eight APS clients underwent VCE, of which 17 (44.7%) were already receiving medical care. Successful follow up with 19 clients showed 14 (73.7%) had care with a PCP 3-6 months after VCE. Of the remaining 5, 2 were still without care, but 3 received care more than 6 months after VCE. These results suggest a significant increase in the percentage of persons who receive medical care after completion of a VCE, though greater improvements can be made. An additional protocol connecting APS with a client’s guardian or family member soon after VCE may improve these numbers, which is particularly important for clients who have active and unmanaged chronic medical illnesses.

